# Quinoline Compound KM11073 Enhances BMP-2-Dependent Osteogenic Differentiation of C2C12 Cells via Activation of p38 Signaling and Exhibits *In Vivo* Bone Forming Activity

**DOI:** 10.1371/journal.pone.0120150

**Published:** 2015-03-19

**Authors:** Seung-hwa Baek, Sik-Won Choi, Sang-Joon Park, Sang-Han Lee, Hang-Suk Chun, Seong Hwan Kim

**Affiliations:** 1 Laboratory of Translational Therapeutics, Pharmacology Research Center, Korea Research Institute of Chemical Technology, Daejeon, 305-600, Republic of Korea; 2 Department of Food Science & Biotechnology, Kyungpook National University, Daegu, 702-701, Republic of Korea; 3 Department of Histology, College of Veterinary Medicine, Kyungpook National University, Daegu, 702-701, Republic of Korea; 4 Alternative Toxicological Methods Research Center, Department of Predictive Toxicology, Korea Institute of Toxicology, Daejeon, 305-600, Republic of Korea; University of California Davis, UNITED STATES

## Abstract

Recombinant human bone morphogenetic protein (rhBMP)-2 has been approved by the FDA for clinical application, but its use is limited due to high cost and a supra-physiological dose for therapeutic efficacy. Therefore, recent studies have focused on the generation of new therapeutic small molecules to induce bone formation or potentiate the osteogenic activity of BMP-2. Here, we show that [4-(7-chloroquinolin-4-yl) piperazino][1-phenyl-5-(trifluoromethyl)-1H-pyrazol-4-yl]methanone (KM11073) strongly enhances the BMP-2-stimulated induction of alkaline phosphatase (ALP), an early phase biomarker of osteoblast differentiation, in bi-potential mesenchymal progenitor C2C12 cells. The KM11073-mediated ALP induction was inhibited by the BMP antagonist noggin, suggesting that its osteogenic activity occurs via BMP signaling. In addition, a pharmacological inhibition study suggested the involvement of p38 activation in the osteogenic action of KM11073 accompanied by enhanced expression of BMP-2, -6, and -7 mRNA. Furthermore, the *in vivo* osteogenic activity of KM11073 was confirmed in zebrafish and mouse calvarial bone formation models, suggesting the possibility of its single use for bone formation. In conclusion, the combination of rhBMP-2 with osteogenic small molecules could reduce the use of expensive rhBMP-2, mitigating the undesirable side effects of its supra-physiological dose for therapeutic efficacy. Moreover, due to their inherent physical properties, small molecules could represent the next generation of regenerative medicine.

## Introduction

A delicate balance between osteoclast-mediated bone resorption and osteoblast-mediated bone formation is necessary for normal bone development and remodeling. Excessive osteoclastic bone resorption and/or reduced bone formation results in bone loss that consequently leads to pathological bone-related disorders, such as osteoporosis, rheumatoid arthritis, periodontal disease, and cancer bone metastasis [1]. These bone-related disorders impact clinical and public health, most importantly due to subsequent fractures. Bone fractures are one of the most common causes of disability and are associated with enormous healthcare expenditures.

**Fig 1:**
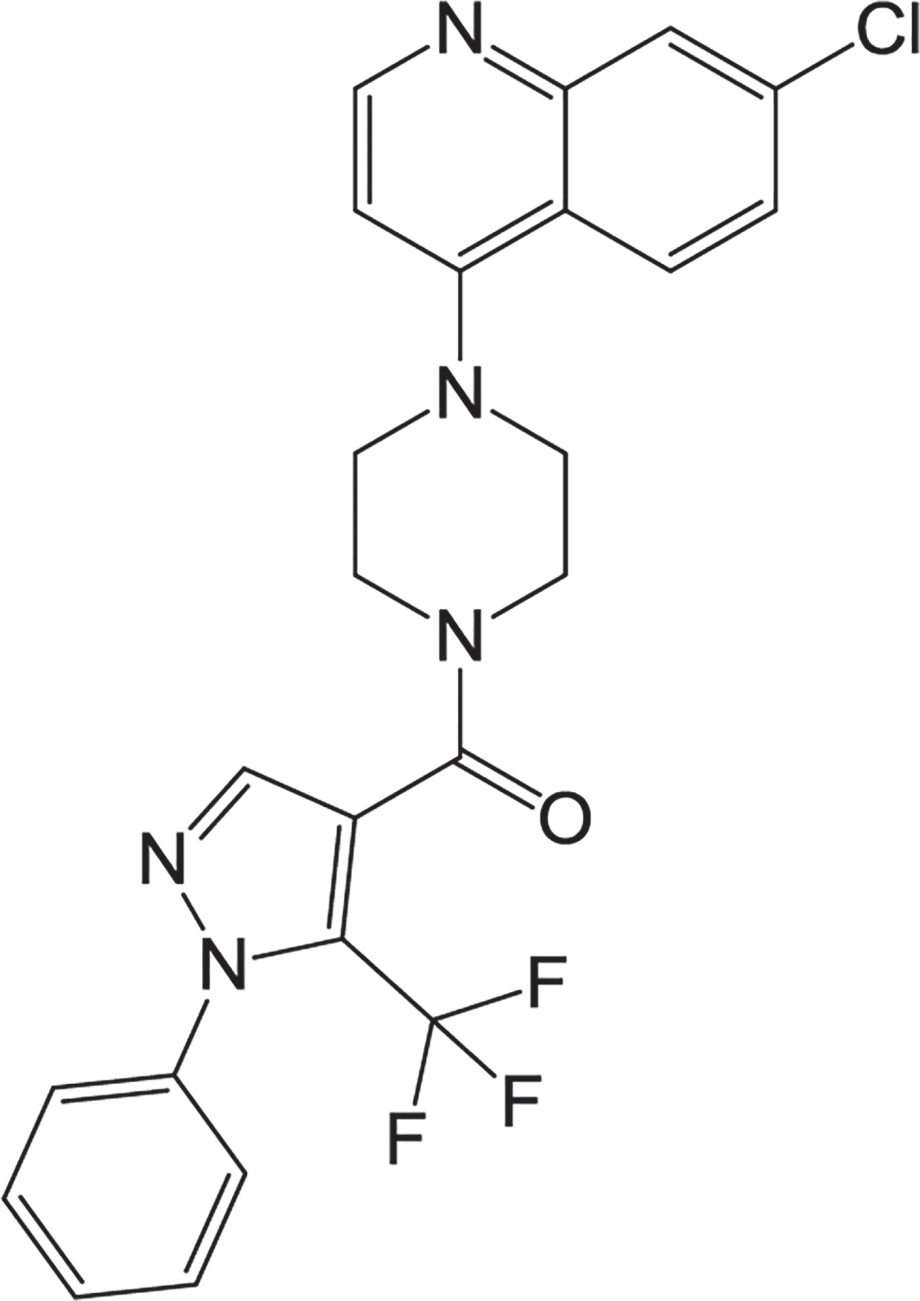
Chemical structure of KM11073.

Most agents that are used to inhibit bone loss are anti-resorptive agents, but the development of anabolic agents for stimulating bone formation is also an area of interest [2,3]. Among FDA-approved anabolic agents, recombinant human bone morphogenetic proteins (rhBMPs) have potential clinical applications in spinal fusion, fracture healing, and dental tissue engineering [4–7]. BMPs play crucial roles in bone formation, repair, and regeneration [8–10]. As one of osteogenic BMP family, BMP-2 strongly triggers the commitment of mesenchymal stem cells into pre-osteoblasts for bone formation and mineralization. rhBMP-2 has been approved by the FDA for application in spinal fusion and the treatment of long bone fractures [7, 11], but its clinical use is limited due to its comparatively expensive cost and severe side effects, among other reasons. Therefore, recent studies have focused on the identification of new effective anabolic small molecules that are less expensive and simple to use [3]. In the previous study, chemical library in Korea Chemical Bank was screened in order to identify anabolic compounds in the BMP-2-mediated osteoblast differentiation model of bi-potential mesenchymal precursor C2C12 cells [12], and finally [4-(7-chloroquinolin-4-yl) piperazino][1-phenyl-5-(trifluoromethyl)-1H-pyrazol-4-yl]methanone (KM11073; Fig. 1) was identified as a BMP-2 enhancer that can accelerate the BMP-2-mediated commitment of C2C12 cells into osteoblasts. Therefore, in the present study, the effect of KM11073 on the commitment of C2C12 cells into osteoblasts was confirmed and potential mechanisms explaining its osteogenic activity investigated.

## Materials and Methods

### Materials

KM11073 was purchased from Maybridge (MO, USA). In this study, 10 mM KM11073 in DMSO was used as a stock solution and diluted with culture medium. Therefore, 0.2% DMSO was used as a vehicle control in all experiments. Recombinant human BMP-2 (rhBMP-2) and noggin were purchased from PeproTech (Seoul, Korea). Ras inhibitor FTI-277, PI3K inhibitor LY294002, Akt inhibitor, and p38 inhibitors (PD169316, SB203581, and SB202190) were purchased from Calbiochem (EMD Biosciences, Inc., La Jolla, CA, USA).

### Cell culture

C2C12 cells were maintained in Dulbecco’s Modified Eagle’s Medium (DMEM, Hyclone) containing 10% fetal bovine serum (FBS), 100 U/ml of penicillin, and 100 μg/ml streptomycin. Cells were seeded and, after 1 day, differentiated by replacing the medium with differentiation medium (DM; DMEM containing 5% FBS and 100 ng/ml rhBMP-2). The medium was changed every 3 days.

### Cell viability assay

Cells were seeded in a 96-well plate at 4 × 10^3^ cells/well. After 24 h, the cells were cultured in DM with serially diluted KM11073 for 1 or 3 days. Cell growth was evaluated in triplicate using Cell Counting Kit-8 (Dojindo Molecular Technologies, ML, USA) according to the manufacturer’s protocol. Absorbance was measured at 450 nm using the Wallac EnVision microplate reader (PerkinElmer, Finland) and the measured absorbance converted to cell number using the standard curve.

### Alkaline phosphatase (ALP) staining and activity assay

ALP, an early biomarker of osteoblastogenesis, was assayed as described previously [13]. Briefly, C2C12 cells (4 × 10^3^ cells/well) were seeded in a 96-well plate, and after 24 h, the medium replaced with DM in the absence or presence of KM11073. The medium was changed every 3 days. After 6 days, the cells were washed twice with PBS, fixed with 10% formalin in PBS for 30 s, rinsed with deionized water, and stained using the Alkaline Phosphatase (ALP) Kit (Sigma). To measure ALP activity, cells were washed twice with PBS and sonicated in lysis buffer (10 mM of Tris–HCl pH 7.5, 0.5 mM of MgCl_2_, and 0.1% Triton X-100). After centrifugation at 10,000 × g for 20 min at 4°C, ALP activity was measured in triplicate in the supernatant using the LabAssay ALP Kit (Wako Pure Chemicals Industries).

### Evaluation of mRNA expression

Primers were designed using an online primer design program [14] (Table 1). Total RNA was isolated and cDNA prepared as described previously [13]. Briefly, total RNA was isolated in C2C12 cells (2 × 10^4^ cells/well in a 24-well plate) using TRIzol reagent (Life Technologies, MD, USA) and the first strand cDNA synthesized using 2 μg of total RNA, 1 μM of oligo-dT_18_ primer, and Omniscript Reverse Transcriptase (Qiagen, CA, USA). SYBR green-based quantitative PCR was performed using the Stratagene Mx3000P Real-Time PCR system and Brilliant SYBR Green Master Mix (Stratagene, CA, USA) as described previously [13]. All reactions were run in triplicate and data analyzed using the 2^−ΔΔCT^ method [15,16]. GAPDH was used as the control gene. Significance was determined with GAPDH-normalized 2^−ΔΔCT^ values. The mRNA levels in zebrafish were evaluated as follows. Five days after fertilization, 20 embryos per well were transferred to a 12-well plate with 2 ml of test solution. After 1 day, total RNA was isolated using TRIzol reagent and the first strand cDNA synthesized using 2 μg of total RNA, 1 μM of oligo-dT_18_ primer, and Omniscript Reverse Transcriptase (Qiagen, CA, USA). SYBR green-based quantitative PCR was performed as described above.

**Table 1:** Primer sequences used in this study.

Target		Forward (5’–3’)	Reverse (5’–3’)
C2C12	*BMP-2*	GCTCCACAAACGAGAAAAGC	AGCAAGGGGAAAAGGACACT
	*BMP-4*	CCTGGTAACCGAATGCTGAT	AGCCGGTAAAGATCCCTCAT
	*BMP-6*	TTCTTCAAGGTGAGCGAGGT	TAGTTGGCAGCGTAGCCTTT
	*BMP-7*	CGATACCACCATCGGGAGTTC	AAGGTCTCGTTGTCAAATCGC
	*BMP-9*	CAGAACTGGGAACAAGCATCC	GCCGCTGAGGTTTAGGCTG
	*ALP*	ATGGGCGTCTCCACAGTAAC	TCACCCGAGTGGTAGTCACA
	*GAPDH*	AACTTTGGCATTGTGGAAGG	ACACATTGGGGGTAGGAACA
Zebrafish	*runx2a*	TTTGAGCGTCAATTCCCAAG	GGTACGGTGGAGGCAGGTAT
	*BMP-2a*	CTCCATCCCAGACGAGGAGT	GCTCCTGAAGAGAACCGGAC
	*BMP-2b*	GTGAGGGTCAGTCGTTCCCT	AGCATGTCGCCTACAGTTCG
	*osteopontin*	ATGATCTGGAGGACGGGAAC	GCTGGGAGAGTCCCTAGCAC
	*ALP*	CGCAATTAAGCAGGGAATCA	CCTGCGTTTACGGATTTTCA
	*GAPDH*	AGAACATCATCCCAGCCTCC	TTGGCAGGTTTCTCAAGACG

### Western blot analysis

Western blot analysis was performed as described previously [13]. Briefly, cells were homogenized in ice-cold buffer consisting 10 mM Tris–HCl (pH 7.5), 150 mM NaCl, 0.05% (v/v) Tween 20, 1 mM PMSF, and one protease inhibitor cocktail tablet (Roche, Germany) and then centrifuged at 10,000 × g for 15 min. Protein concentrations were determined using the BCA protein assay kit (Pierce, IL, USA) and denatured proteins separated and transferred to PVDF membrane (Millipore, USA). Membranes were incubated with TBST buffer (10 mM Tris–HCl pH 7.5, 150 mM NaCl, 0.1% Tween 20) with 5% nonfat dry milk and then incubated with diluted primary antibodies (1:1,000) overnight at 4°C. The antibodies used in this study were purchased from Cell Signaling (MA, USA) or Santa Cruz Biotechnology, Inc. (Santa Cruz, CA, USA). Following the primary antibody reactions, the membranes were washed with TBST buffer three times (15 min each) and then probed with diluted secondary antibodies (1:5,000) for 1 h. Next, the membranes were washed three times (15 min each) and developed with SuperSignal West Femto Maximum Sensitivity Substrate (Pierce Biotechnology) using the LAS-3000 luminescent image analyzer (Fuji Photo Film Co., Ltd., Japan). Each experiment was performed at least three times, and the figures showed the results from one representative experiment. Image J software-based quantification of the detected band was carried out and the relative, normalized ratio between phosphorylated protein and the protein itself was presented in the figures.

### Alizarin red S-based zebrafish skeleton development

The development of zebrafish skeletons were visualized by alizarin red S according to the Standard Protocol was approved the Institutional Animal Care and Use Committees of Chungnam National University (CNU-00393). As described previously [17], zebrafish embryos were placed in a 24-well plate (9 embryos per well) 5 days after fertilization and maintained in 1 ml of buffered medium (sea salt, 0.06 mg/l) containing KM11073. The medium was changed every day. Seven days after fertilization, the embryos were fixed in 2% paraformaldehyde for 1 h and washed three times with PBS containing 0.1% Tween 20 (PBST) at 10-min intervals. Next, the embryos were treated with 1 ml of alizarin red S staining buffer (pH 4.2) to stain the formed bone overnight. The embryos were washed two times with 25% glycerol (in 1% KOH) and bleaching solution (1% KOH and 3% H_2_O_2_ in deionized water) at room temperature until pigmented cells removed after approximately 15 min. When the pigmented cell removal was complete, the embryos were washed three times with 25% glycerol (in 1% KOH) at 10-min intervals and the embryos treated successively with KOH solutions containing 25% glycerol, 50% glycerol, and 80% glycerol.

### *In vivo* murine calvarial bone formation assay

*In vivo* bone formation experiments were carried out according to the Ethics Guidelines of the Korea Research Institute of Chemical Technology (Protocol ID No. 7D-M5). The protocol was approved by the Institutional Committee of the Korea Research Institute of Chemical Technology (Approval No. 2014-7D-04-05). The surgery for *in vivo* bone formation experiments was performed under anesthesia, and all efforts were made to minimize animal suffering. Briefly, *in vivo* bone-forming activity was evaluated using lyophilized collagen sponges as described previously [13]. After anesthesia with the intraperitoneal injection of 1.2% avertin (600 μl/mouse), collagen sponges loaded with 5 μl of DMSO or KM11073 (2.5 or 5 mM) were implanted over the calvarial bones of 5-week-old male ICR mice (n = 5 per group; Central Lab Animal, Seoul, Korea). When mice were monitored daily, no suffering by operation was observed. Three weeks after drug implantation, the calvariae were harvested after cervical dislocation, fixed in 4% paraformaldehyde, decalcified in 12% EDTA, embedded in paraffin, and sectioned. Sections were deparaffinized through graded xylene washes, dehydrated in a graded series of ethanol washes, and stained with hematoxylin and eosin (H&E).

### Statistical analysis

Significance was determined by the Student’s *t* test and differences were considered significant when *p* < 0.05.

## Results

**Fig 2:**
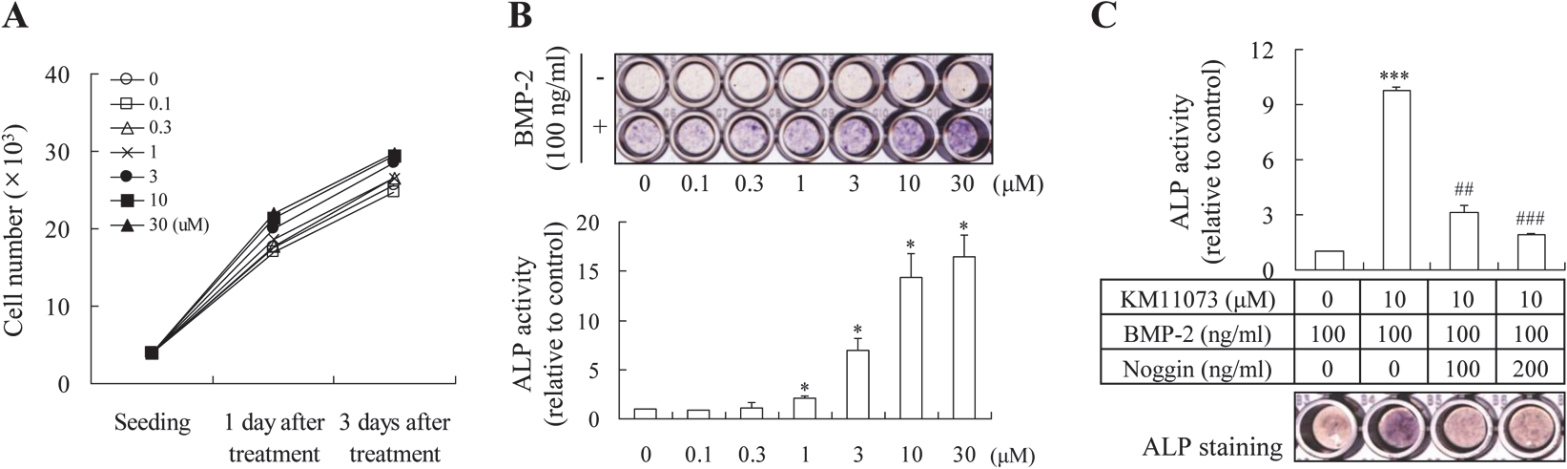
KM11073 enhanced BMP-2-induced osteoblast differentiation in C2C12 cells. Cell viability was assayed 1 and 3 days after treatment with KM11073 (*A*). Effect of KM11073 on BMP-2-stimulated ALP induction. Cells (4 × 10^3^ cells/well) were cultured in a 96-well plate for 1 day and then the medium replaced with DMEM containing 5% FBS and KM11073 in the presence or absence of rhBMP-2 (100 ng/ml). The medium was changed every 3 days. On differentiation day 6, ALP staining and its activity were assayed *(B)*. Effect of noggin on KM11073-mediated enhancement of BMP-2-stimulated ALP induction. Osteogenesis was enhanced by KM11073 in the presence of BMP-2 on differentiation days 0 and 2, and then noggin was treated on differentiation day 4. On differentiation day 6, ALP staining and its activity were assayed (*C*). *** *p* < 0.001 compared to the BMP-2-treated group; ^##^ *p* < 0.01, ^###^ *p* < 0.001 compared to the group treated with BMP-2 and KM11073.

The enhancing effect of KM11073 on the BMP-2-mediated commitment of C2C12 cells into osteoblasts was evaluated by visualizing ALP expression and measuring its activity. At non-cytotoxic concentrations (≤ 30 μM; Fig. 2A), KM11073 induced the expression of ALP in a dose-dependent manner in the presence of BMP-2 (Fig. 2B). Consistent with this result, KM11073 significantly enhanced BMP-2-stimulated ALP activity in a dose-dependent manner (Fig. 2B). In the absence of BMP-2, KM11073 did not induce ALP expression, suggesting that the osteogenic activity of KM11073 relies on BMP-2 signaling. This finding was confirmed by the post-treatment of noggin; when noggin was post-treated alone on differentiation day 4 after BMP-2 and KM11073 treated on differentiation day 0 and 2, noggin dose-dependently and significantly inhibited the ALP activity induced by both BMP-2 and KM11073 (Fig. 2C).

The inhibition of KM11073-enhanced ALP induction by noggin also suggested the involvement of endogenous BMP induction in the osteogenic activity of KM11073. Therefore, we evaluated the effect of KM11073 on the expression of osteogenic BMPs (Table 2). ALP expression and osteogenic BMPs were significantly induced by BMP-2 alone, and KM11073 significantly enhanced the BMP-2-stimulated mRNA levels of BMP-2, BMP-6, and BMP-7, as well as ALP, but not BMP-4 and BMP-9. In the absence of BMP-2, KM11073 did not induce the mRNA expressions of BMP-2, BMP-6, and BMP-7 (S1 Table).

**Table 2:** Involvement of p38 inhibitors in the KM11073-mediated enhancement of BMP-2-stimulated induction of osteogenic genes.

mRNA	*BMP-2*	*BMP-4*	*BMP-6*	*BMP-7*	*BMP-9*	*ALP*
Control	1.00 ± 0.13	1.00 ± 0.08	1.00 ± 0.13	1.00 ± 0.14	1.00 ± 0.17	1.00 ± 0.43
BMP-2	3.32 ± 0.96*	2.54 ± 0.16**	3.07 ± 0.54*	3.93 ± 0.52*	3.25 ± 0.06*	2.44 ± 0.14
BMP-2 + KM11073	10.44 ± 1.12** ^,^ ^#^	2.10 ± 0.31*	12.52 ± 2.62** ^,^ ^#^	12.52 ± 2.62** ^,^ ^#^	2.36 ± 0.87	9.82 ± 0.45* ^,^ ^##^
BMP-2 + KM11073 + PD169316	5.19 ± 0.08** ^,^ ^†^	1.98 ± 0.39*	2.73 ± 0.24* ^,^ ^†^	3.74 ± 0.31**	3.80 ± 0.21**	2.42 ± 0.81^††^
BMP-2 + KM11073 + SB202190	3.48 ± 0.54* ^,^ ^†^	1.63 ± 0.14* ^,^ ^#^	2.52 ± 0.10* ^,^ ^††^	3.96 ± 0.02*	3.53 ± 0.04** ^,^ ^#^	1.55 ± 0.20^#^ ^,^ ^††^
BMP-2 + KM11073 + SB203580	6.78 ± 2.62*	2.04 ± 0.36*	3.15 ± 0.63* ^,^ ^†^	3.60 ± 1.08*	3.44 ± 0.37*	2.81 ± 0.03^††^

Cells were treated with each inhibitor for 2 h and then incubated with BMP-2 (100 ng/ml) and KM11073 (10 μM) for 3 days. The mRNA expression levels were evaluated by quantitative real-time PCR. Fold changes relative to each gene level in the control are presented as mean ± standard deviation

* *p* < 0.05

** *p* < 0.01 (compared to the control)

^#^ *p* < 0.05

^##^ *p* < 0.01 (compared to the group treated with BMP-2)

^†^ *p* < 0.05

^††^ *p* < 0.01 (compared to the group treated with BMP-2 + KM11073).

**Fig 3:**
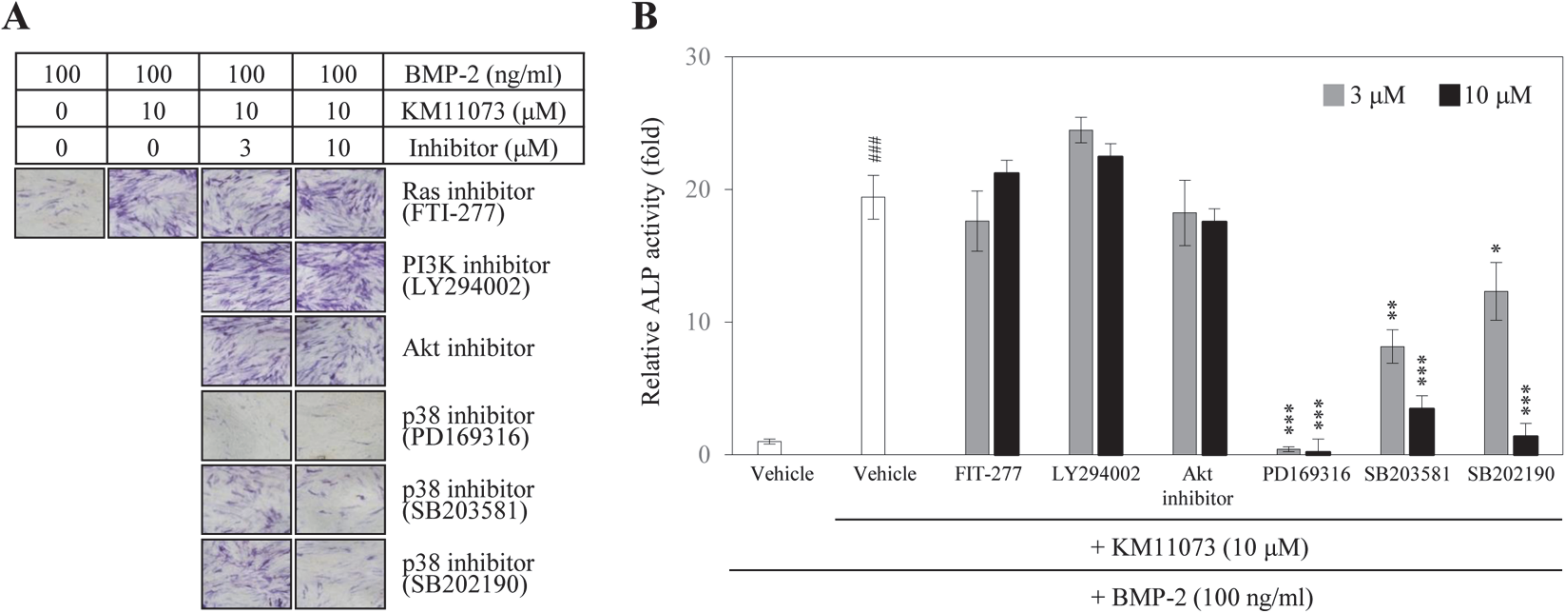
Involvement of p38 in the KM11073-mediated enhancement of BMP-2-stimulated ALP induction. In a 96-well plate, cells (4 × 10^3^ cells/well) were treated with each inhibitor for 2 h and then treated with BMP-2 and KM11073. After 3 days, the cells were treated with each inhibitor. On differentiation day 6, ALP staining (A) and its activity (B) were assayed. ^###^ *p* < 0.001 compared to the BMP-2-treated group; * *p* < 0.05, ** *p* < 0.01, *** *p* < 0.001 compared to the group treated with BMP-2 and KM11073.

Next, in order to investigate the underlying mechanism explaining the osteogenic activity of KM11073, a pharmacological inhibition study was performed. The KM11073-enhanced ALP induction in the presence of BMP-2 was strongly inhibited by p38 inhibitors, but not inhibitors of Ras, phosphatidylinositol 3-kinase (PI3K), or Akt (Fig. 3).

**Fig 4:**
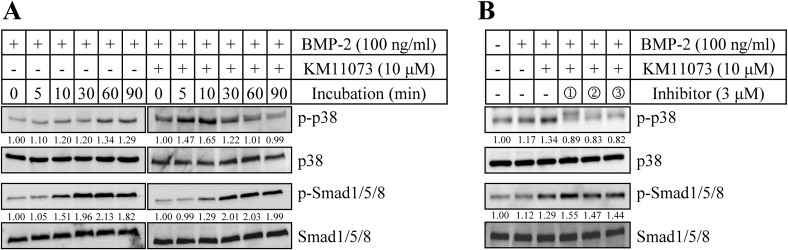
Effect of KM11073 on the activation of p38. Cells (1 × 10^5^ cells/well) were cultured in a 6-well plate for 1 day and then incubated with DMEM containing 5% FBS in the presence or absence of BMP-2 and/or KM11073 (*A*). Inhibitory effects of p38 inhibitors (1, SB202190; 2, PD169316; 3, SB203580) on the activation of p38 by BMP-2 and KM11073. Western blot analysis was performed with protein samples prepared with cells treated with each inhibitor for 30 min and then incubated with BMP-2 and KM11073 for 30 min (*B*). The relative, normalized ratio between phosphorylated protein and the protein itself was presented.

The involvement of the p38 signaling pathway in KM11073-mediated ALP induction was confirmed by Western blot analysis. BMP-2 induced the phosphorylation of p-38 1 h after its treatment, and this induction was triggered more quickly by the addition of KM11073; 5 min after the addition of KM11073, p38 phosphorylation was observed in C2C12 cells (Fig. 4A). However, the induction was strongly inhibited by p38 inhibitors, suggesting the involvement of p38 activation in the osteogenic action of KM11073 (Fig. 4B). In addition, KM11073-enhanced expression of BMP-2, BMP-6, and BMP-7 was significantly inhibited by p38 inhibitors (Table 2). In the absence of BMP-2, KM11073 did not induce the mRNA expressions of BMP-2, BMP-6, and BMP-7 (S1 Table).

The involvement of Smad activation in the anabolic action of KM11073 via p38 activation was evaluated by Western blot analysis. As shown in Fig. 4A, BMP-2 induced the phosphorylation of Smad1/5/8, but its induction was not enhanced or accelerated by KM11073. Moreover, the phosphorylation of Smad1/5/8 induced by BMP-2 and KM11073 was not dramatically inhibited by p38 inhibitors, suggesting that the osteogenic action of KM11073 is Smad-independent (Fig. 4B). In the absence of BMP-2, the protein expressions of Smad and p38, and their phosphorylations were not changed by KM11073 or inhibitor alone (S1 Fig.).

**Fig 5:**
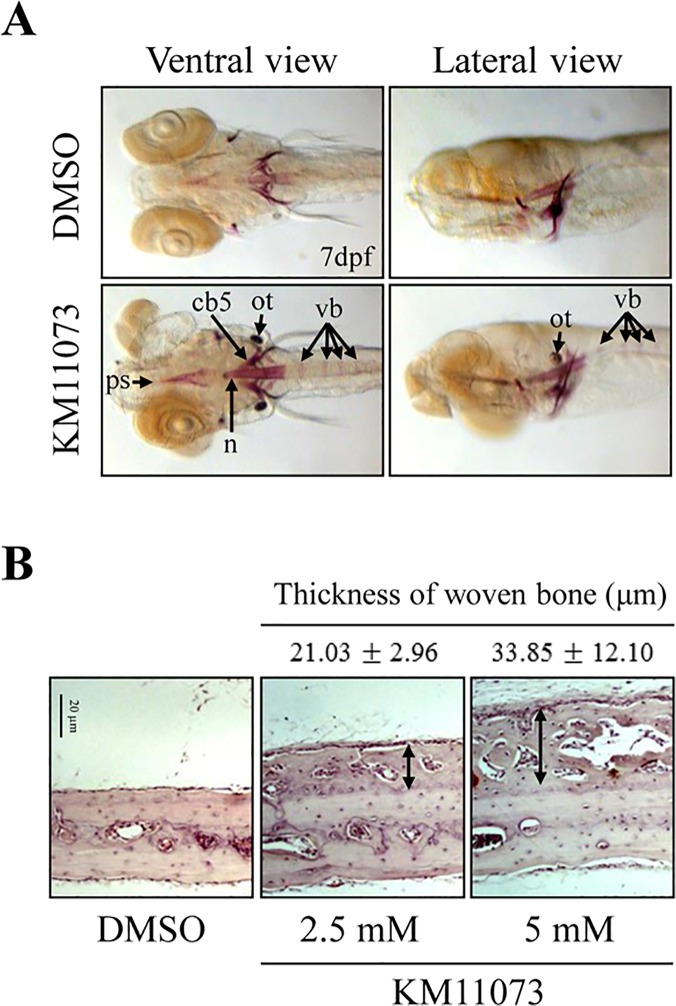
Evaluation of the *in vivo* osteogenic activity of KM11073 in zebrafish and mouse calvariae. Five days after fertilization, zebrafish were treated with KM11073 (1 μM) for 2 days and then fixed and stained with alizarin red S. The parasphenoid (ps), notochord (n), ceratobranchial 5 (cb5), otolith (ot), and vertebrae (vb) are indicated with arrows (*A*). Collagen sponges soaked in 5 μl of 2.5 or 5 mM KM11073 were placed onto mouse calvarial bones. After 3-week implantation, the mice were sacrificed. Calvarial bones were removed, fixed, decalcified, embedded in paraffin, and sectioned. Sections were stained with H&E and photographed at 200 × magnification. Arrows indicate the thickness of newly formed woven bones (*B*). The thickness of newly formed woven bones was quantified compared to the scale bar.

The anabolic activity of KM11073 was further evaluated in two *in vivo* models, the zebrafish skeletal development model and the mouse calvarial bone formation model. KM11073 (1 μM) enhanced the development of the parasphenoid, notochord, ceratobranchial 5, otolith, and vertebrae (Fig. 5A). The bone-forming activity of KM11073 was also confirmed by evaluating the mRNA expression levels of genes related to osteogenesis in zebrafish (Table 3). When larvae at 5 days post fertilization (dpf) were incubated with KM11073 (1 μM) for 1 day, the mRNA levels of Runx2a, BMP-2a, OP, and ALP were significantly induced by KM11073. In addition, the enhancing effect of KM11073 on *in vitro* mineralization in osteoblast cells was also observed (S2 Fig.).

**Table 3:** Osteogenesis-related gene expression in zebrafish.

mRNA	*runx2a*	*bmp2a*	*bmp2b*	*osteopontin*	*ALP*
Control	1.00 ± 0.34	1.00 ± 0.06	1.00 ± 0.33	1.00 ± 0.15	1.00 ± 0.13
KM11073	11.69 ± 0.88*	4.83 ± 1.63*	3.75 ± 1.52	4.20 ± 0.60*	3.90 ± 0.91*

At 5.0 dpf, larvae were treated with KM11073 (1 μM), and after 1 day the mRNA levels were evaluated by quantitative real-time PCR. Fold changes relative to each gene level in the control are presented as mean ± standard deviation.

* *p* < 0.05

** *p* < 0.01 (compared to the control).

The *in vivo* mouse calvarial bone formation assay also revealed the bone-forming activity of KM11073; H&E staining showed that implanted collagen sponges soaked with KM11073 induced woven bone formation over the periosteum of the calvarial bones (Fig. 5B). The thickness of newly formed woven bone in mice treated with 2.5 and 5 mM of KM11073 was 21.03 and 33.85 μm, respectively.

## Discussion

An increased risk of complications and adverse events has been suggested for patients receiving rhBMP-2 in spinal fusion, and safety concerns about its clinical application have emerged, including a greater apparent risk of new malignancy with higher doses of rhBMP-2 [18]. Because BMPs are expensive to produce, small molecules that enhance their potential to induce bone formation would be a cost-effective alternative that reduces the BMP-mediated adverse effects [19,20].

In this study, we found an enhancing effect of KM11073 on the BMP-2-mediated commitment of C2C12 cells into osteoblasts. The stimulatory effect of KM11073 on ALP induction was not observed in the absence of BMP-2, suggesting that its osteogenic activity relies on BMP-2 signaling. This suggestion was confirmed by strong inhibition of the KM11073-mediated enhancement of ALP induction in the presence of BMP-2 by noggin post-treatment. Noggin is a well-known BMP antagonist and its functional role in bone formation was identified in a silencing study; the knockdown of noggin has been shown to enhance osteoblastogenesis of BMP-responding cells *in vitro* and rhBMP-2-induced new bone formation *in vivo* [21].

We also identified a BMP-2-dependent osteogenic action of KM11073 via accelerated activation of p38. Recently, the activation of Akt and MAP kinases, which are downstream in the Ras-PI3K signaling pathway, has been shown to enhance osteoblastogenesis and bone formation by inducing BMP-2 gene expression [22]. However, in this study, pharmacological inhibition revealed that p38 inhibitors exhibit a dose-dependent inhibitory effect on ALP expression induced by KM11073 in the presence of BMP-2, suggesting the involvement of p38 activation in the osteogenic action of KM11073. The involvement of p38 activation in osteoblast differentiation has been reported in several studies [13,23,24].

BMP-2 triggers osteogenic signaling through the action of its signaling mediator, Smad. The phosphorylation and translocation of Smad has been shown to induce the expression of osteoblastogenesis-related genes [25–28]. In this study, BMP-2 induced the phosphorylation of Smad, but its induction was not enhanced or accelerated by KM11073. Furthermore, the phosphorylation of Smad was not inhibited by p38 inhibitors. These results suggest that the BMP-2-dependent osteogenic action of KM11073 relies on the activation of p38 but not Smad. BMP-2-induced osteogenic differentiation through a Smad1/5/8 phosphorylation-independent pathway has been reported [29], and conversely, activation of the Smad pathway has been shown to be independent of BMP-2 signaling [30]. Distinct BMP-receptor complexes have been suggested to initiate Smad-dependent or Smad-independent signaling [31].

The induction of osteogenic BMPs is also important in the BMP-2-mediated commitment of mesenchymal stem cells into osteoblasts. BMP-2 enhanced the expression of other osteogenic BMP genes during osteoblast differentiation [32], and the gene transfer of BMP-2 and -7 induced rapid bone formation and increased the expression of endogenous BMP-4 [33]. Here, BMP-2 significantly induced the mRNA expression of all BMPs tested in this study, and KM11073 enhanced the BMP-2-induced mRNA expression of BMP-2, BMP-6, and BMP-7, but not BMP-4 or BMP-9. Although the osteogenic effect differs among the BMPs and among the types of cell exposed to these proteins [34], BMP-6 can also induce osteoblastic differentiation of mesenchymal stem cells [35,36]. The osteogenic activity of BMP-7 in C2C12 cells has also been reported [37]. KM11073-enhanced expression of osteogenic BMP-2, BMP-6, and BMP-7 could further augment the osteogenic activity of KM11073. This possibility may also be supported by the results that the post-treatment of noggin strongly inhibited the KM11073-enhanced induction of ALP. In addition, KM11073-enhanced expression of BMP-2, BMP-6, and BMP-7 was significantly inhibited by p38 inhibitors, confirming the involvement of p38 activation in the osteogenic action of KM11073.

Furthermore, the *in vivo* bone-forming activity of KM11073 was confirmed in two *in vivo* models, the zebrafish skeletal development model and the mouse calvarial bone formation model [38]. The pathway for bone formation in mammal has been suggested to be conserved during the development of the skeleton in zebrafish [39]. Runx2 (runt-related transcription factor) is essential for osteoblast differentiation during skeletal development in mammals. In an expression analysis of bone markers in zebrafish larvae, *runx2a*, one of the zebrafish homologs of mouse *Runx2*, was expressed in the early stage of bone development [39,40]. Also, both bmp2a and bmp2b in zebrafish have been cloned and are expressed 10 to 36 h after fertilization [41,42]. Here, in addition to markers of osteogenesis (osteopontin and ALP), *runx2a* and *bmp2a* mRNA, but not *bmp2b*, were significantly induced by KM11073 in zebrafish larvae.

In summary, KM11073 exhibited BMP-2-dependent *in vitro* osteogenic activity via the activation of p38 signaling. In addition, the activities of quinolin analogues to enhance BMP-2-mediated ALP activity were also found (S2 Table). Therefore, the combination of rhBMP-2 with osteogenic small molecules could reduce the use of expensive rhBMP-2, which mitigates undesirable side effects caused by its supra-physiological dose for therapeutic efficacy. Furthermore, the *in vivo* osteogenic activity of KM11073 *per se* also suggests its potential single use for bone formation. The inherent physical properties of small molecules minimizing the limitations of using biologics suggests that they represent the next generation of regenerative medicine [43].

## Supporting Information

S1 Fig Effect of KM11073 or inhibitor on the expression and activation of Smad and p38 in C2C12 cells. Effect of KM11073 or inhibitor on the expression and activation of Smad and p38 was evaluated by Western blot analysis. Briefly, C2C12 Cells (1 × 10^5^ cells/well) were cultured in a 6-well plate for 1 day and then incubated with DMEM containing 5% FBS in the presence or absence of KM11073 and each p38 inhibitor for 30 min.
(TIF)

S2 Fig KM11073 enhanced the mineralization in mouse primary osteoblast cells. (A) The primary calvarial pre-osteoblasts differentiated with ascorbic acid (50 μg/ml), β-glycerophosphate (10 mM), and BMP-2 (50 ng/ml) in the absence or presence of KM11073 (0.3 μM). Medium was changed every 3 days, and the mineralization was visualized by alizarin red S staining on day 9. (B) Deposited alizarin red S was dissolved with 10% cetylpyridinium (Sigma-Aldrich) for 15 min at room temperature and quantified by a multiplate reader (Envision) at 560 nm.
(TIF)

S1 Supporting Information Materials and Methods. (DOCX)

S1 Table Effect of KM11074 and inhibitors on mRNA expression of BMPs. (DOCX)

S2 Table Enhancing effect of quinolin analogues on the BMP-2-induced ALP expression in C2C12 cells. (DOCX)
