# Supplementary figures and images for: Quinoline Compound KM11073 Enhances BMP-2-Dependent Osteogenic Differentiation of C2C12 Cells via Activation of p38 Signaling and Exhibits In Vivo Bone Forming Activity
Source: PLoS One. 2015 Mar 19;10(3):e0120150. doi: 10.1371/journal.pone.0120150 (PMC4366212; doi:10.1371/journal.pone.0120150)

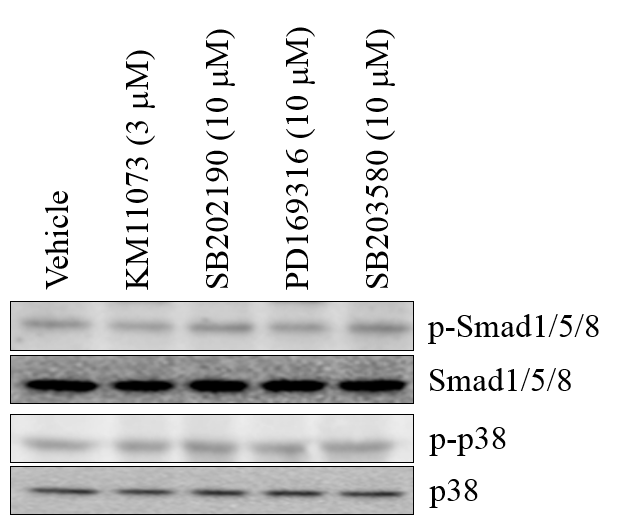

Supplement: S1 Fig — Effect of KM11073 or inhibitor on the expression and activation of Smad and p38 was evaluated by Western blot analysis. Briefly, C2C12 Cells (1 × 10^5^ cells/well) were cultured in a 6-well plate for 1 day and then incubated with DMEM containing 5% FBS in the presence or absence of KM11073 and each p38 inhibitor for 30 min. (TIF) [file pone.0120150.s001.tif]

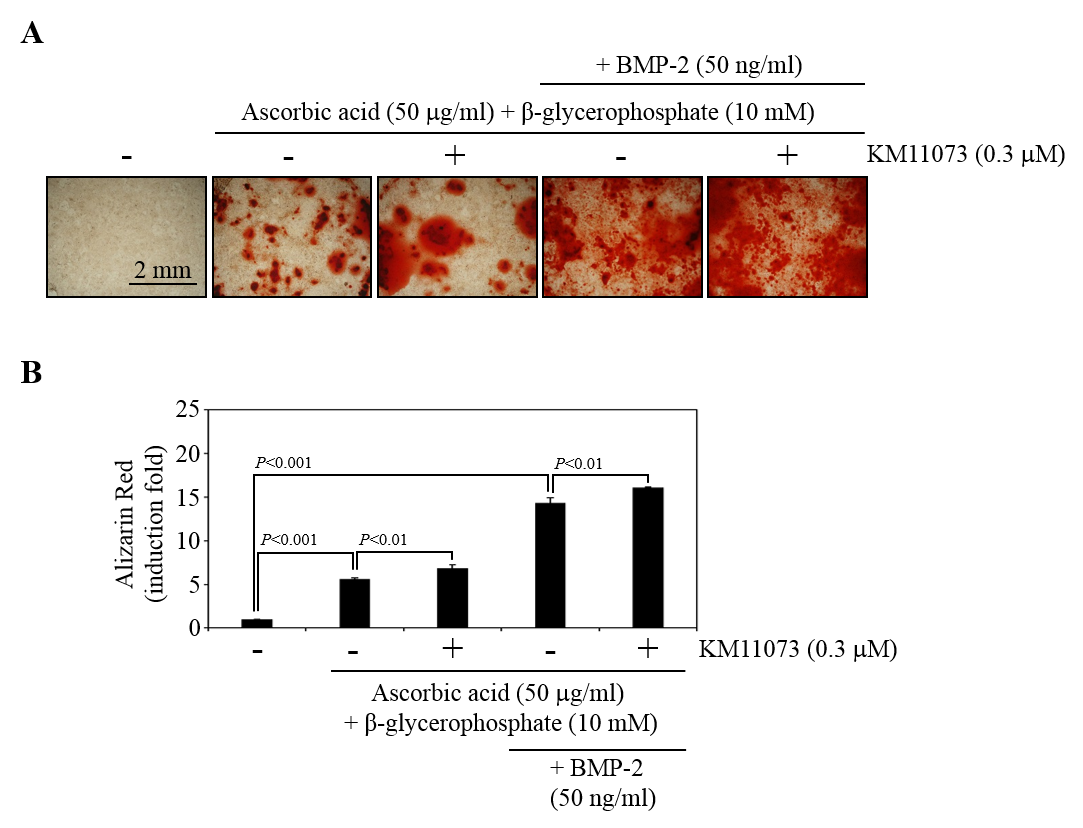

Supplement: S2 Fig — (A) The primary calvarial pre-osteoblasts differentiated with ascorbic acid (50 μg/ml), β-glycerophosphate (10 mM), and BMP-2 (50 ng/ml) in the absence or presence of KM11073 (0.3 μM). Medium was changed every 3 days, and the mineralization was visualized by alizarin red S staining on day 9. (B) Deposited alizarin red S was dissolved with 10% cetylpyridinium (Sigma-Aldrich) for 15 min at room temperature and quantified by a multiplate reader (Envision) at 560 nm. (TIF) [file pone.0120150.s002.tif]
