# Supplementary material for: Quinoline Compound KM11073 Enhances BMP-2-Dependent Osteogenic Differentiation of C2C12 Cells via Activation of p38 Signaling and Exhibits In Vivo Bone Forming Activity
Source: PLoS One. 2015 Mar 19;10(3):e0120150. doi: 10.1371/journal.pone.0120150 (PMC4366212; doi:10.1371/journal.pone.0120150)
Supplement: S1 Supporting Information — (DOCX) [file pone.0120150.s003.docx]

**Supporting Information**

**Quinoline compound KM11073 enhances BMP-2-dependent osteogenic differentiation of C2C12 cells via activation of p38 signaling and exhibits *in vivo* bone forming activity**

Seung-hwa Baek^1,2,*^, Sik-Won Choi^1,*^, Sang-Joon Park^3^, Sang-Han Lee^2^, Hang-Suk Chun^4,5^, Seong Hwan Kim^1,**^

^1^Laboratory of Translational Therapeutics, Pharmacology Research Center, Korea Research Institute of Chemical Technology, Daejeon 305-600, Republic of Korea; ^2^Department of Food Science & Biotechnology, Kyungpook National University, Daegu 702-701, Republic of Korea; ^3^Department of Histology, College of Veterinary Medicine, Kyungpook National University, Daegu 702-701, Republic of Korea; ^4^Alternative Toxicological Methods Research Center, Department of Predictive Toxicology, Korea Institute of Toxicology, Daejeon 305-600, Republic of Korea; ^5^Department of Biology, Chungnam National University, Daejeon 305-510, Republic of Korea

**Materials and Methods**

***Mineralization in mouse primary osteoblast cells***

According to the Ethics Guidelines in Korea Research Institute of Chemical Technology (Protocol ID No. 7D-M4) and the approval by Institutional Committee (Approved No. 2014-7D-04-04), newborn ICR mouse calvaria were digested by several addition of 0.1% collagenase (Gibco) and 0.2% dispase (Gibco) for obtaining mouse primary pre-osteoblast cells. The supernatants after the first digestion were discarded, and the primary pre-osteoblast cells were collected by the centrifugation of the supernatants obtained from the second to the fourth digestion, amplified with α-MEM (Gibco) containing 10% FBS (as called to growth medium) and frozen for future use. For differentiation, the isolated primary pre-osteoblasts were seeded in a 96-well plate and after become confluent, the medium was replaced with the differentiation medium (growth medium containing 50 μg/ml ascorbic acid, 10 mM β-glycerophosphate and 50 ng/ml BMP-2). The differentiation medium was changed every 3 days. For visualizing the mineralization, cells were rinsed with PBS on the differentiation day 9, fixed in 10% buffered formalin, rinsed with deionized water, and stained with 2% Alizarin red S (Applichem). For quantification, stained alizarin red was dissolved with 10% cetylpyridinium (Sigma-Aldrich) for 15 min at room temperature. Then, the extraction was measured by a multiplate reader (Envision) at 560 nm.
