# Supplementary material for: Quinoline Compound KM11073 Enhances BMP-2-Dependent Osteogenic Differentiation of C2C12 Cells via Activation of p38 Signaling and Exhibits In Vivo Bone Forming Activity
Source: PLoS One. 2015 Mar 19;10(3):e0120150. doi: 10.1371/journal.pone.0120150 (PMC4366212; doi:10.1371/journal.pone.0120150)
Supplement: S1 Table — (DOCX) [file pone.0120150.s004.docx]

| mRNA | *BMP-2* | *BMP-4* | *BMP-6* | *BMP-7* | *BMP-9* |
| --- | --- | --- | --- | --- | --- |
| Control | 1.00 ± 0.20 | 1.00 ± 0.01 | 1.00 ± 0.51 | 1.00 ± 0.20 | 1.00 ± 0.21 |
| KM11073 | 1.26 ± 0.29 | 1.36 ± 0.18^*^ | 0.91 ± 0.23 | 1.28 ± 0.03 | 1.27 ± 0.13 |
| PD169316 | 1.71 ± 1.02 | 0.97 ± 0.83 | 1.59 ± 0.02 | 1.66 ± 0.34 | 1.44 ± 0.31 |
| SB202190 | 1.38 ± 0.47 | 1.09 ± 0.89 | 1.41 ± 0.01 | 1.21 ± 0.15 | 1.31 ± 1.06 |
| SB203580 | 1.27 ± 0.04 | 0.70 ± 0.36 | 0.83 ± 0.04 | 1.19 ± 0.04 | 0.96 ± 0.05 |

Cells were treated with compound (10 μM) for 3 days, and then the mRNA expression levels were evaluated by quantitative real-time PCR. Fold changes relative to each gene level in the control are presented as mean ± standard deviation. ^*^ *p* < 0.05 (compared to the control)
