# Supplementary material for: Quinoline Compound KM11073 Enhances BMP-2-Dependent Osteogenic Differentiation of C2C12 Cells via Activation of p38 Signaling and Exhibits In Vivo Bone Forming Activity
Source: PLoS One. 2015 Mar 19;10(3):e0120150. doi: 10.1371/journal.pone.0120150 (PMC4366212; doi:10.1371/journal.pone.0120150)
Supplement: S2 Table — (DOCX) [file pone.0120150.s005.docx]

| Structure | 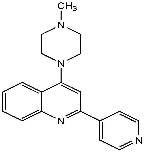 | 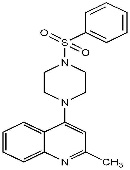 | 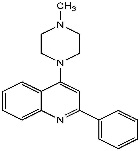 | 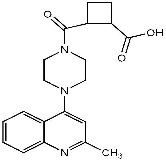 | 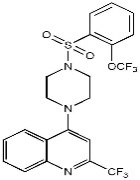  (KM11073) | 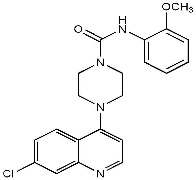 | 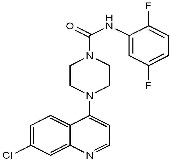 | 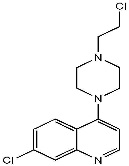 | 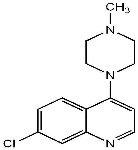 | 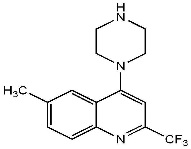 |
| --- | --- | --- | --- | --- | --- | --- | --- | --- | --- | --- |
| % of ALP activity (BMP-2 alone, 100 %) | 341 | 299 | 290 | 258 | 228 | 209 | 206 | 179 | 112 | 110 |

Cells (4 × 10^3^ cells/well) were cultured in a 96-well plate for 1 day and then the medium replaced with DMEM containing 5% FBS and each quinolin compound (3 μM) in the presence of rhBMP-2 (100 ng/ml). On differentiation day 3, ALP activity was assayed. Relative % of ALP activity of compound compared to that of BMP-2-treated group alone are presented.
